# Direct Seeding and Transplanting Influence Root Dynamics, Morpho-Physiology, Yield, and Head Quality of Globe Artichoke

**DOI:** 10.3390/plants10050899

**Published:** 2021-04-29

**Authors:** Daniel I. Leskovar, Yahia A. Othman

**Affiliations:** 1 Texas A&M AgriLife Research and Extension Center, Texas A&M University, Uvalde, TX 78801, USA; ya.othman@ju.edu.jo; 2 Department of Horticulture and Crop Science, The University of Jordan, Amman 11942, Jordan

**Keywords:** *Cynara cardunculus*, root length, minirhizotron, yield, SPAD, chlorogenic acid

## Abstract

The objective of this two-year field study was to assess the influence of stand establishment methods (direct seeding or transplanting) on root growth dynamics, shoot morphology, leaf physiology, yield, and quality of globe artichoke (*Cynara cardunculus*). Three artichoke cultivars were evaluated, ‘Green Globe Improved’ (GGI), ‘Imperial Star’ (IS), and ‘Romolo’ (ROM). Plants established with the transplanting method had higher mean root length intensity (*L*a), root length, and root surface area as compared to plants established by direct seeding. The topsoil (0–20 cm) had on average higher *L*a, root length, and root surface area than deeper soil profiles. Transplanted plants had higher plant shoot width and leaf area index (LAI) chlorophyll content index (SPAD) than direct seeded plants at the vegetative stage in 2015. The improvement of root and shoot growth in transplants (compared to direct seeding) also resulted in higher (*p* < 0.05) marketable yield (21.1 vs. 19.9 ton ha^−1^ in 2015 and 18.3 vs. 13.7 ton ha^−1^ in 2016). Additionally, 46–50% of the total yield occurred during the first 30 days of harvest in the transplanting method compared to 13–38% for direct seeding. No significant differences were found between planting methods or cultivars in leaf-level gas exchange (photosynthesis, stomatal conductance, and transpiration) and cynarin concentration in the marketable heads. Although chlorogenic acid was similar in both establishment methods in 2015, direct seeding had higher concentration in 2016. Comparing cultivars, GGI had higher root length, surface area, root volume, and earlier and higher marketable yield than ROM. However, ROM had higher mean root length intensity (*L*a; total root length per specific area in soil profile) than GGI in both growing seasons. This study showed significant and consistent improvements in root and shoot traits, and yield for transplants as compared to direct seeded plants.

## 1. Introduction

Farming systems, nitrogen management, cultivar selection, and planting methods such as direct seeding and transplanting are cultural strategies that greatly influence root and shoot growth, yield, and fruit quality in high-value vegetable crops [1,2,3,4]. In bell pepper (*Capsicum annuum*), earlier studies comparing direct seeding with containerized transplants produced in nurseries showed significant effects on growth (leaf area and shoot weight) and developmental stages in the field [5]. For certain crop species (e.g., onion, rice) established at high planting density, direct seeding has been considered a cost-effective method of stand establishment since transplanting would require the additional costs for raising the transplants in nurseries and high labor costs of transplanting [6,7]. However, consideration of the high seed cost and lower seedling survival in direct seeding is important, especially for high-value vegetable crops that are typically hybrids instead of open pollinated cultivars. The applicability and success of direct seeding depends on seed quality, knowledge of appropriate sowing times and rates, seedbed preparation, field germination, emergence, and resource competition from emerging weeds [8]. The main constraints on stand success from direct seeding that could lead to high mortality are poor germination, uneven stands, transient or persistent environmental stresses (episodes of droughts, desiccation events, floods, excessive heat), diseases, weed competition, and limited nutrients [7]. Artichoke (*Cynara cardunculus*) fields established with the transplanting method typically have less weed pressure and disease problems and higher yield and head uniformity as compared to direct seeding [9]. Transplant quality can also be improved during the nursery stage by optimizing the nitrogen nutrition of the growing substrate in the tray cells, a practice that was shown to mitigate the transplanting shock during stand establishment of globe artichoke [3].

Transplanting is a reliable method to improve growth and achieve earliness and higher yield, as reported for several crops, including onion (*Allium cepa*), rice (*Oryza sativa*), bell pepper, tomato (*Solanum lycopersicum*), and watermelon (*Citrullus lanatus*) [1,5,6,10]. Onion established by transplanting resulted in higher yield (36.3 t ha^−1^) than direct seeding (19.5 t ha^−1^) and matured earlier (104 days) compared to the direct seeded (135 days) method [6]. However, the transplanting date for onion (early, mid-, or late planting) is critical to produce higher yield [11]. Transplanted rice had higher leaf chlorophyll content index (SPAD), N concentration, total root length, and total root tip number than direct seeded plants [12]. In southern England, a suitable combination of cultivar and age of transplants shortened the growth period and enabled corn plants to meet the thermal time requirement for a grain harvest [1]. In tomato, transplants had earlier and higher yield than direct seeded plant [5]. In smallholder farms in South Africa, the adoption of mature transplants over direct seeding to establish corn plants has significantly increased survival and decreased the damage caused by birds during early developmental stages [2]. A comparative review of using direct seeding and seedling plantings in restoration projects revealed that the successful survival percentage of transplants was three times higher than direct seeding, especially when using larger seeds (*Albizia, Acacia, Phyllanthus*, and *Ocotea*) [7].

Root traits such as root length, root surface area, and length intensity (*L*a; total root length per specific area in the soil profile) can significantly affect plant morphology and leaf physiology, as reported in globe artichoke and olive crops [3,4,13]. Planting methods can also affect early root development, root and shoot biomass allometric partitioning, fruit development, and marketable yields [2,14,15]. For example, in bell pepper, transplanted plants had higher basal root dry weight percentage (transplants, 81% vs. direct seeded plants, 25%) and smaller lateral roots (15% vs. 57%) and taproot (4% vs. 18%). While direct seeded plants sustained more balanced root, stem, leaf, and fruit dry matter partitioning than transplants, the latter exhibited higher and earlier yields [15].

Plant internal factors, such as leaf anatomy and morpho-physiology, can significantly affect growth and development, as well as yield [16]. Photosynthesis (*Pn*) normally increases rapidly from leaf emergence, reaching maximum values at full leaf expansion. Physiological performance, including *Pn* and stomatal conductance (*g*s), can increase due to higher leaf thickness and contents of chlorophyll (a + b) and palisade parenchyma because those leaf variables help to capture a greater amount of light [16,17]. However, the effects of those internal factors in artichoke have not been well studied in direct seeded and transplanted plants.

Globe artichoke is a popular Mediterranean crop rich in antioxidant compounds such as chlorogenic acid, dicaffeoylquinic acids, and cynarin, which are known to be beneficial for human health [18,19]. In addition to genotype, cultural practices and farming systems have been shown to affect the content of chlorogenic acid, with ranges from 60–600 (µg g^−1^), and cynarin from 2 to 20 (µg g^−1^) [20,21,22,23]. In organic farming, head chlorogenic acid increased by 31% and cynarin by 12% compared to those grown in conventional fields [19]. Shinohara et al. [24] also found that irrigation practices were more effective than the selection of N rates to optimize artichoke crop yield and head quality (chlorogenic acid). To date, the impact of planting method in root growth dynamics and head quality of artichoke cultivars has not been investigated. Accordingly, the objective of this study was to determine the differential expression of shoot and root traits (root length, surface area, volume, diameter, and *L*a) in relation to the most common planting methods (transplant vs. direct seeding). We hypothesize that when compared to direct seeded plants, artichoke transplants will have a differential root growth pattern, with more biomass allocation directed towards root length, especially during the vegetative developmental stage and for early cultivars; these responses will in turn translate into improvements in early and total marketable yield. The findings of this study contribute to new knowledge centered on the importance of root traits to improve crop growth and productivity of artichoke transplants.

## 2. Results

Table 1 shows the ANOVA and mean separation (LSD) for *L*a as affected by planting method, cultivar, and soil depth for two growing seasons, 2015–2016. Plants analyzed in 2015 were transplanted (or sown) in October 2014 while the 2016 plants were transplanted (or sown) in November 2015. Since the study considered the annual system the 2015-started plants were terminated at the end of the cycle and thus not analyzed in 2016. Mean *L*a was significantly different for planting method, cultivar, and soil depth during 2015 and 2016. Transplants had higher mean *L*a in both years (2015–2016) as well as the overall means. Root length values for direct seeded plants never exceeded those of transplants across months and over the study period 2015–2016. However, *L*a response varied with cultivars. For example, ROM and IS had higher root *L*a in March and April 2015 while GGI had higher *L*a than IS in July 2015 and 2016 (Table 1). The main *L*a values (>90%) for the three tested artichoke cultivars were within 0–80 cm of soil depth. A soil depth of 0–20 cm had the highest mean *L*a in 2015 while the 20–40 and 40–60 *L*a were higher than the other soil depths in 2016. However, the overall mean (2015–2016) for *L*a was similar across the 0–60 cm soil depths.

**Figure 1:**
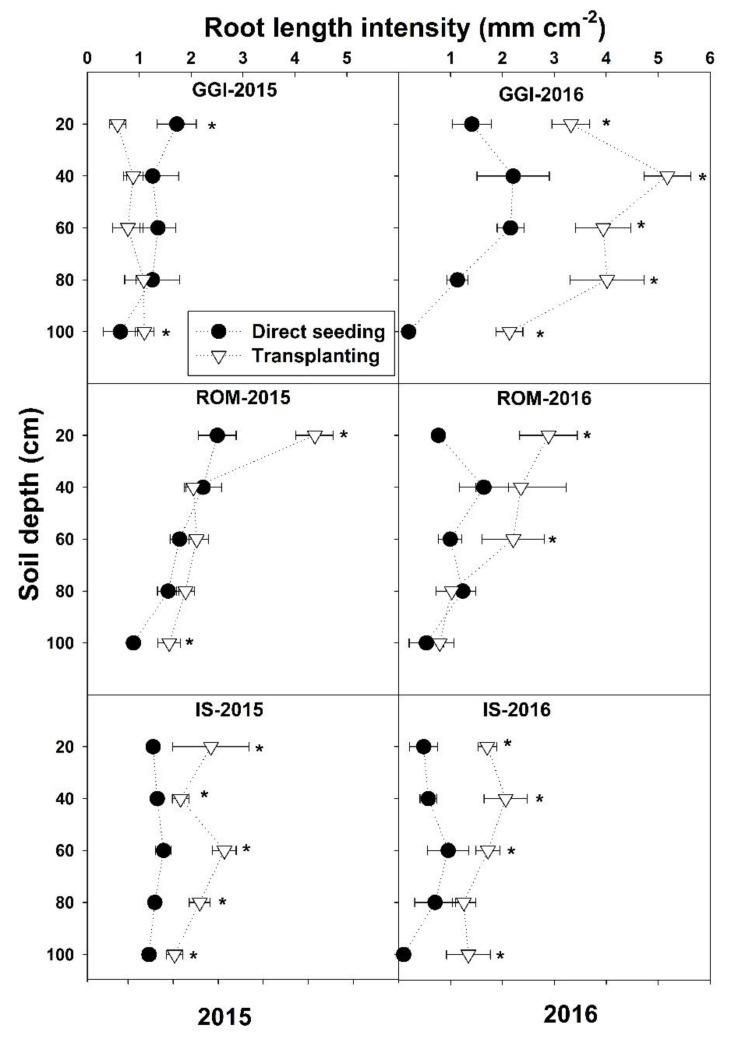
Mean root length intensity at different soil depths for artichoke cultivars (Green Globe Improved (GGI), Romolo (ROM) and Imperial Star (IS)) as influenced by establishment method (direct seeding and transplanting) during 2015 and 2016 seasons. Data were collected using minirhizotron root system. Asterisk (*) within each soil depth represents significant differences between treatments at *p* < 0.05.

There was a significant planting method and cultivar interaction for *L*a across months and over the study period 2015–2016, except in May 2015 (Table 1 and Figure 1). The planting method and cultivar interactions revealed that, in both years, *L*a values from the transplanting method were higher or similar (never lower) to direct seeding across the soil depths (0–100) and cultivars (GGI, ROM, IS), except for GGI at a 0–20 cm soil depth in 2015 (Figure 1).

**Figure 2:**
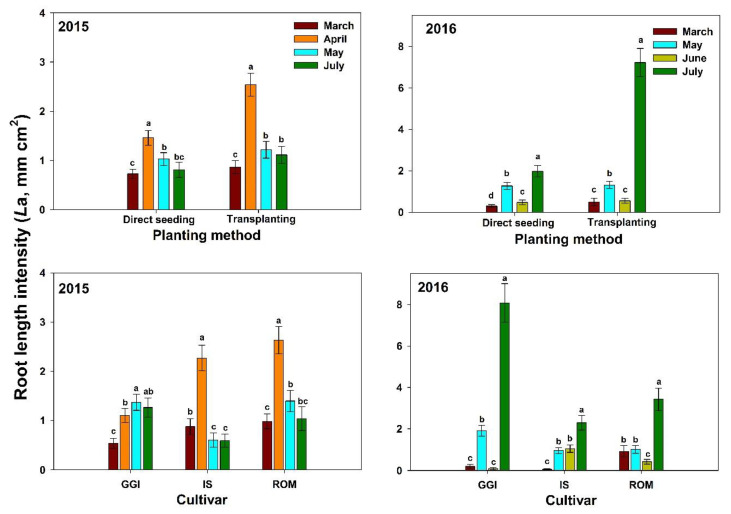
Mean root length intensity at soil depth 0–100 cm for artichoke cultivars (Green Globe Improved (GGI), Romolo (ROM) and Imperial Star (IS)) as influenced by planting method (direct seeding and transplanting) during 2015 and 2016 seasons. Data were collected using the minirhizotron root system. Bars within the same harvesting date followed by different letters are significantly different at *p* < 0.05.

In both years (2015–2016), artichoke plants had the lowest *L*a (0–100 cm soil depth) during the vegetative stages (March, about four months after planting) across planting methods (direct seeding and transplanting) and over cultivars (GGI, IS, ROM) (Figure 2). Both direct seeding and transplanting *L*a values were low in March, peaked during the harvesting period (April), and decreased thereafter (July) in 2015. However, both planting methods had the highest *L*a after harvest (July) in 2016 (Figure 2). Roots in 2016 were longer (about 40%)than those in 2015. Similar trends were noticed for tested cultivars in both years, except for GGI in 2015.

**Figure 3:**
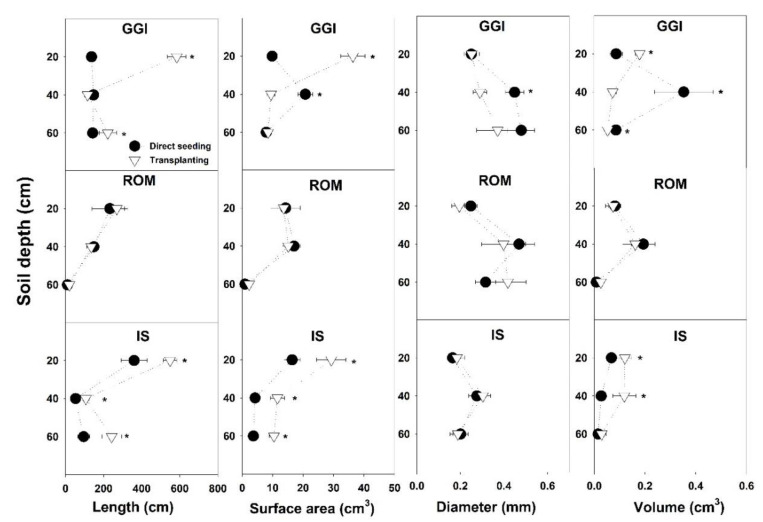
Root components of artichoke cultivars (Green Globe Improved (GGI), Romolo (ROM) or Imperial Star (IS)) as influenced by planting method (direct seeding and transplanting) at three soil depths during the harvesting period, July 2015. Asterisk (*) within each soil depth represents significant differences between treatments at *p* < 0.05.

Soil cores sampling in July 2015 revealed that root component values, specifically length and surface area from the transplanting method, were higher than those from direct seeding (Table 2). The GGI cultivar had consistently higher root length, surface area, and volume than ROM. While the artichoke plants from a 0–20 cm soil depth had higher root length and surface area than the 20–40 or 40–60 cm depths, root volumes from these combined lower depths (20–60 cm) were higher than at the topsoil layer, 0–20 cm (Table 2). Planting method × cultivar showed that transplanting resulted in longer or similar (never shorter) root length than direct seeding across soil depths (0–60 cm) and cultivars (GGI, ROM, IS) (Figure 2). Similarly, root surface area in transplanted artichokes was larger than in direct seeded plants, except for GGI at a soil depth of 20–40 cm. For root diameter and volume, the planting method × cultivar interactions were inconsistent or not significant across soil depths. For example, at 20–40 cm, GGI cultivar root volume from the direct seeding treatment was larger than for transplants, while IS from direct seeding had lower values at the same soil depth (Figure 3).

Parameters used as a measure of plant morphology (width and height) and their main physiological processes (leaf area index (LAI), chlorophyll content index (SPAD), photosynthesis (*Pn*), stomatal conductance (*g*s), and transpiration (*E*)) were determined at the vegetative and harvesting stages in both growing seasons. In 2015, plant width, height, and LAI at the harvesting stage as well as SPAD at the vegetative stage were higher in transplanted vs. directly seeded artichokes (Table 3). In 2016, plant size at the vegetation stage was larger for transplanting than the direct seeding method. However, cultivar responses were not significant or inconsistent across the study period. In addition, *Pn*, *g*s, and *E* for direct seeding and transplanting were statistically similar in both years (2015, 2016) and across cultivars (data not presented). Photosynthesis values ranged from 19–21 µmol m^−2^ s^−1^ in 2015 and from 28–30 µmol m^−2^ s^−1^ in 2016 (numerically higher in 2016); *g*s was about 0.4 mol m^−2^ s^−1^ in 2015 and 0.5 mol m^−2^ s^−1^ in 2016; *E* was about 2.5 mmol m^−2^ s^−1^ in 2015 and 5.5 mmol m^−2^ s^−1^ in 2016. However, gas exchange (*Pn*, *g*s, and *E*) values for 2015 were about 50% lower than 2016 across cultivars and over planting methods (direct seeding and transplanting) though the differences between years (2015 vs. 2016) were not statistically analyzed.

**Figure 4:**
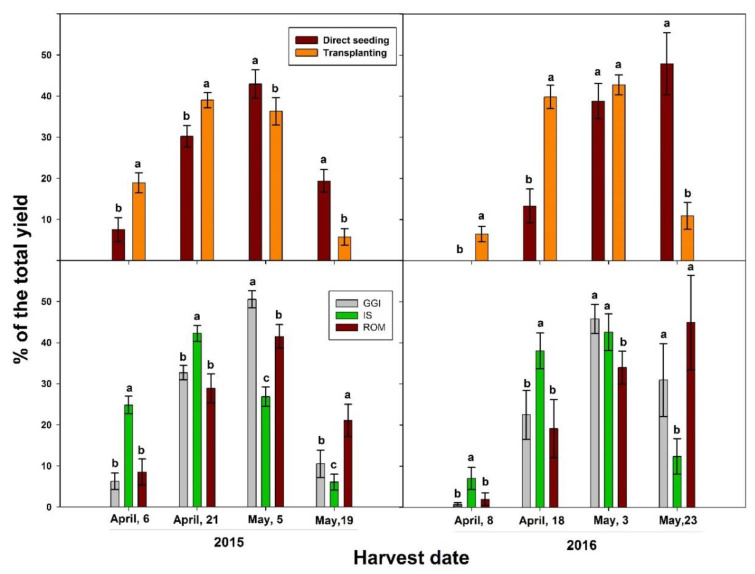
Percentage of the total yield across harvesting periods (2015, 2016) of artichoke cultivars (Green Globe Improved (GGI), Romolo (ROM), and Imperial Star (IS)) as influenced by planting methods, direct seeding, and transplanting. Bars within the same harvesting date followed by different letters are significantly different at *p* < 0.05.

The transplanting method significantly increased artichoke total yield in both 2015 and 2016 growing seasons, when compared to direct seeding (Table 4). In addition, the transplanting method resulted in earlier harvesting and yield in both growing seasons (Figure 4). In 2015, the percentage of heads from early harvests out of the total yield was 58% for transplanting and 38% for direct seeding. Similarly, the percentage was 46% for transplanting and 13% for direct seeding in 2016. Considering cultivars, IS had the earliest production and the highest yield percentage in the first month, while the ROM cultivar had the latest head production across the study period, 2015–2016 (Figure 4). Specifically, more than 60% of the total marketable yield for IS occurred between April 6 and 21, 2015 while 65% of ROM yield occurred between May 5 and May 19 of the same growing season. However, there were no significant differences between treatments (transplant vs. direct seeding) in head quality (chlorogenic acid and cynarin), except for chlorogenic acid in 2016. In that year, direct seeding had higher chlorogenic acid concentration in the head than those from the transplanting method (Table 4). In 2015, GGI had higher yield than ROM and IS, while in 2016 GGI and IS had higher yield than ROM (Table 4). Higher yield for GGI was associated with a significantly lower content of chlorogenic acid concentration, especially in 2015 (Table 4). However, there were no significant differences in cynarin concentration between cultivars and across planting methods in both years.

## 3. Discussion

### 3.1. Root Growth Dynamics

Comparing both plant establishment systems across the study period data from Table 1 and Table 2 showed that transplants had higher mean root *L*a, length, and surface area as compared to direct seeded plants. The expression of root traits is highly associated with plant growth and productivity, as demonstrated in bell pepper and young olive seedlings [13,15]. In rice, it has been shown that increases in root components such as root biomass, length, and density, as well as root oxidation activity and root zeatin + zeatin riboside content during the early and mid-growing season, led to higher grain yield [25]. In artichoke seedlings, higher root trait components, such as root length and surface area during the transplanting stage, increased total marketable yield [3,9]. Early research in tomato and pepper has demonstrated that root architecture in direct seeded and transplanted plants are quite different. While direct seeded plants develop a vertical strong taproot in non-compacted soils, transplanted seedlings develop a distinctive root system with typically more basal roots derived from the root–hypocotyl transition zone, which is caused by the early modification of the taproot in the containers; these changes also lead to more uniform growth and higher crop yields compared to direct seeding [9,15,26,27].

Physiologically, the function of diverse root growth components is critical for the establishment of young plants, especially just after planting [25]. At this early stage, the root systems of newly planted seedlings might be inadequate to rapidly supply enough water to shoots, leading to transplant shock [3,9,28]. The ability of seedlings to overcome post-planting stress is affected by several factors such as root architecture (size and distribution), root–soil interaction, and root hydraulic conductivity [28]. In this study, most root traits were highly expressed in the topsoil (0–20 cm) (Table 1 and Table 2). Artichoke *L*a, root length, and root surface area in the topsoil were greater than the lower soil profiles (Table 1 and Table 2). An earlier study in bell pepper by Leskovar et al. [14] found that transplants and direct seeded pepper plants had greater root mass in the 0–10 cm topsoil than in the 10–20 cm soil depth in a sandy soil (150 and 100%, respectively).

### 3.2. Growth, Physiology, Yield, and Head Quality

Across the study period, the results described in Table 3 show an overall increase in plant size for transplants as compared to direct seeding plants. Rice plants established by transplanting had higher *Pn*, *g*s, number of panicles per square meter, seed setting rate, and grain yield, and a smaller number of tillers per plant at the early growth stage and a maximum quantum yield of PSII (*F_v_/F_m_*) compared to direct seeded plants [29]. Higher shoot width and height as well LAI in transplanted plants can be attributed to a larger root system (root length and surface area, Table 3 and Table 4). In lettuce (*Lactuca sativa*), cultivars with large root systems increased nitrogen use efficiency and displayed higher growth rates, leading to higher yields than those cultivars with smaller roots [30]. Higher mean *L*a in 2016 (compared to 2015) was coupled with higher LAI (shoot canopy) at the harvesting stage. This increase in LAI and *L*a in 2016 could be attributed to higher rainfall received by plants that year as compared to the 2015 growing season. In 2015, the total rainfall and irrigation applied to artichoke plants was 630 mm (490 rainfall + 140 irrigation), while in the 2016 growing season, the total rainfall and applied irrigation was 787 mm (687 rainfall + 100 irrigation). In addition, higher soil moisture in 2016 led to higher SPAD (vegetation stage) and gas exchange and consequently higher LAI compared to 2015. Interestingly, the transplanting method reduced applied water in a sugar beet (*Beta vulgaris*) field by about 24% and evapotranspiration by 25% as compared with direct seeding [31].

The observed increase in root and shoot growth and higher yield by the transplanting method compared to direct seeding in artichoke confirms previous results in other crops. In rice, transplanted plants had higher yield than direct seeded in both local and high-yielding cultivars [10]. Tomato transplants had higher survival rate, leaf number, and yield, due to more possible harvests than direct seeded plants [32]. Due to the advanced seedling development, transplants exhibit greater fruit sink demand during the reproductive development than direct seeded plants [14]. Considering the significant and consistent improvement in root components (*L*a, length, and surface area), shoot size, and early and total marketable yield from transplants across the study period (2015–2016), this establishment method could offer significant benefits to artichoke farmers over direct seeding. In both years, 46 to 50% of the total yield was harvested in the first month for transplants, while 13 to 38% in the same period for direct seeded plants (Figure 4). These results agreed with others reported for onion, tomato, and pepper [5,6,15]. A comparative response study of direct seeded and transplanted maize (*Zea mays*) to N fertilization (0, 120, 180, 240, and 300 kg N ha^−1^) showed that transplants reached the flowering stage 11 to 15 days earlier and had higher yield than plants established by direct seeding. It is well known that earliness is a trait highly dependent on the cultivar of choice. In our earlier study, that was the case of for IS, an artichoke cultivar classified as early blooming, while the GGI cultivar is considered to be late blooming [33]. In the present study, more than 50% of the IS yield was harvested in the first month of the harvesting period (2015 and 2016). Interestingly, GGI head production was earlier than ROM (Figure 4) in both years. In fact, 25–45% of ROM yield was harvested in the last week of the harvesting period, while GGI late yield was about 11% and 30% in the latest May harvests of 2015 and 2016, respectively.

Although yield (treatments mean) in 2015 was about 28% higher than 2016, root and shoot growth was lower compared to 2015, though the differences between years (2015 vs. 2016) were not statistically analyzed. In the 2016 growing season, *Pn* and LAI at harvest were about 40% higher than in 2015 (Table 1 and Table 4). Higher biomass in the 2016 year, when plants received more than 157 mm of rainfall water than in 2015, might have led to changes in the source–sink relationship, directing more investment of assimilates towards shoot growth to compensate for the larger canopy. We speculate that a higher supply of water in 2015 might have induced the artichoke plants to invest more in forming heads (stronger sinks) than in biomass of the canopy.

Globe artichoke is a valuable crop and a rich source of antioxidants, such as phenolic acids, flavonoids, and cynarin, which have been used for therapeutic effects [18,19,34,35]. Plants produce reactive oxygen species (ROS) in stress conditions (e.g., drought) and to detoxify ROS, the antioxidants and flavonoids play a key role in protecting plants from abnormal abiotic stresses [36,37]. Significant increases in shoot and reproductive growth have been normally coupled with reductions in nutrient and protein concentrations in tissues due to dilution effects [21,23,38]. In this study, this dilution effect was noticed in the content of chlorogenic acid in 2016 (Table 4), where the significant increase (34%) in artichoke yield from the transplanting method produced heads with reduced (54%) chlorogenic acid. However, cynarin levels from the transplanting and direct seeding heads were statistically similar.

## 4. Materials and Methods

### 4.1. Site Description

**Figure 5:**
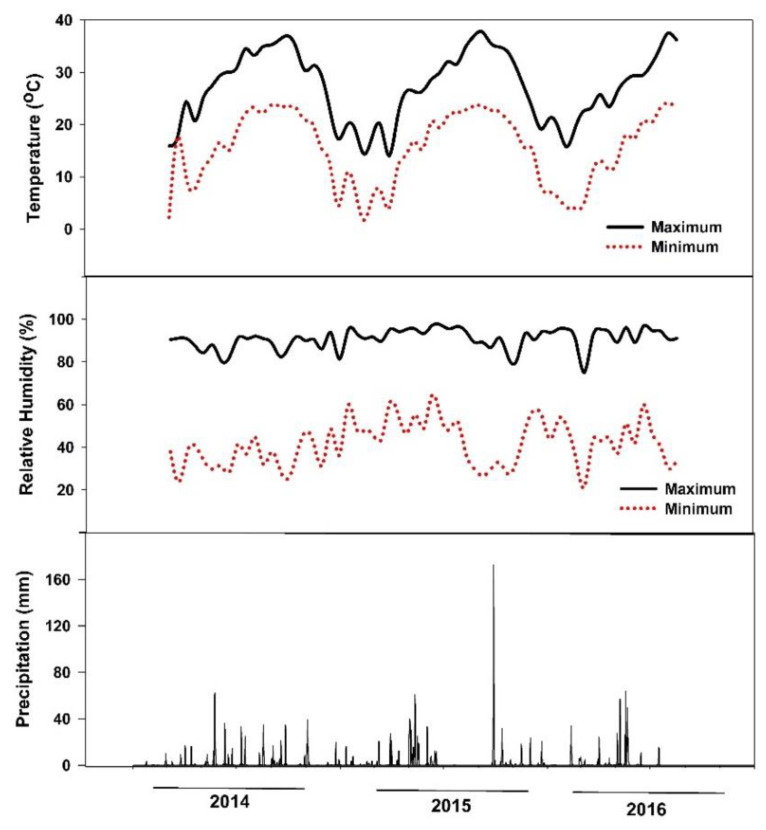
Maximum and minimum air temperature, air relative humidity, and precipitation of the study site at Uvalde, Texas, during the experimental period (October 2014–August 2016).

A two-year field study was conducted at the Texas A&M AgriLife Research and Extension Center at Uvalde, Texas (long. 29°12′57.6″ N, lat. 99°45′21.6″ W) from October 2014 to August 2016. The soil was a clay type (hyperthermic Aridic Calciustolls of the Uvalde series) with the following chemical properties: pH 8.0, EC 0.6 dS m^−1^, P 55 mg kg^−1^, K 810 mg kg^−1^, Ca^+2^ 12,939 mg kg^−1^, Mg^+2^ 333 mg kg^−1^, S 29 mg kg^−1^, Na 50 mg kg^−1^, and nitrate-N 59 mg kg^−1^. In the 2015 growing season, the mean growing temperature was 23 °C, relative humidity 64%, and total rainfall 490 mm, while in 2016 the mean temperature was 21 °C, relative humidity 67%, and total rainfall 687 mm (Figure 5). The plant hardiness zone for the Uvalde site location is 8. Average annual extreme minimum temperatures range from −12.2 to −6.7.

### 4.2. Stand Establishment and Cultivar Treatments

We evaluated two planting methods, direct seeding and transplanting, on three artichoke cultivars, Green Globe Improved (GGI), Imperial Star (IS), and Romolo (ROM). GGI and IS (Big Heart Seed Co, Brawley, California) are open pollinated cultivar types with green to light purple heads, while ROM is a contemporary hybrid cultivar (Big Heart Seed Co, Brawley, California) with predominantly purple heads.

For the transplanting treatment, artichoke seeds of the three cultivars were sown in polystyrene Speedling trays (one seed per cell) containing 128 cells (3.2 × 3.2 cm square and 6.4 cm deep) and placed in a germination chamber for 4 days in darkness inside an incubator chamber set at 20 °C. A 3:1 peatmoss:perlite growing medium was used and only initial watering was required during the incubation period. Then, seedlings were transferred to greenhouse conditions (temperature 23 ± 2 °C, humidity 60 ± 5%) and grown for 7 weeks before field planting. Direct seeding and transplanting were performed simultaneously in the open field on October 28, 2014 (first growing season) and on November 23, 2015 (second growing season). Plants started in 2014 were abandoned after harvest in 2015, and completely new plants were prepared for analysis in 2016. Both establishment and cultivar treatments were planted in separate blocks, each consisting of three beds 1.5 m apart, using a single row per bed at a spacing of 0.9 m between plants. Beds were laid out with a black plastic mulch to reduce weed pressure and soil evaporative water losses. The outside rows were used as buffers and the middle row was used for growth measurements and harvests. For the direct seeded treatment, three seeds per hole were seeded in the field and thinned to one plant after six weeks to be comparable with the transplanting method.

### 4.3. Field Management

In each growing season, N-P-K fertilizers were applied to reach a total rate of 150 N, 100 P and 100 K kg ha^−1^. Fertilizers (4N-4.4P-8.3K and 32N-0P-0K) were applied in 3 split doses each year. The first dose (20% of total fertilizers) was applied the third week after transplanting, the second dose (40%) at the 8-leaf stage, and the third (40%) prior to the beginning of the harvest stage. In both seasons, irrigation was established by a subsurface drip system placed in the middle of the bed at a 15 cm depth. In the 2015 growing season, the number of irrigations was 10 (total irrigation was 140 mm, 10–15 mm per irrigation) while in 2016 the number of irrigations was 7 (total irrigation was 100 mm, 10–15 mm per irrigation). The total rainfall and irrigation for the 2015 growing season was 630 mm (490 rainfall + 140 irrigation) and 787 mm (687 rainfall + 100 irrigation) for 2016. Overall, the total amount of water received by plants was between 630 and 787 mm across the study period. Gibberellic acid (GA_3_, 4%, CP Bio, Inc., Chino, CA) was sprayed twice at 20 mg L^−1^, the first application at the 4th leaf stage and the second 10 d thereafter. Esfenvalerate (Asana XL, 8.4% by weight, DuPont, Wilmington, DE, USA) at 70 mL ha^−1^ was applied to control cucumber beetle (*Diabrotica undecimpunctata*) and cut worms during the vegetative stage. During early head development till harvest, calcium (5%) and zinc (5%) (Tracite, Helena Chemical Co., Fresno, CA, USA) were applied weekly to prevent head atrophia, a physiological disorder typically associated with calcium deficiency [3].

### 4.4. Root Measurements

Root measurements were conducted four times during each growing season following the procedures of Othman and Leskovar [13] and Sharma et al. [39]. Root measurements were taken using the minirhizotron technique with acrylic tubes of 182 cm in length and 50.8 mm in diameter. Minirhizotron tubes were installed 30 cm away from the seedlings at a 45° angle from the vertical using a trailer-mounted Giddings hydraulic probe (5-TS MODEL-MGSRTS, Giddings Machine Co., Windsor, CO, USA). The aboveground 30 cm of the minirhizotron tubes were painted with a double layer of black (inside) and white (outside) paint to prevent light penetration and the top end of each tube was covered with a PVC end cap for further light and moisture protection. In both growing seasons, five specific soil depth (SSD) ranges were used, 0–20, 20–40, 40–60, 60–80, and 80–100 cm. The SSD of each photo was calculated following the procedure of Rasmussen and Thorup-Kristensen [40] as *cos* (45°) × tube depth of the photo. As the minirhizotron tubes were installed at a 45° angle from the vertical line, four minirhizotron images were collected within each interval depth and the four values from each soil depth interval were averaged to one value prior to statistical analysis. A microscope camera system (Bartz Technology Corporation, Carpinteria, CA, USA) was used to capture the root pictures from the upper interface of the tube and the soil [39,40]. The total area represented in each image was 3.24 cm^2^. Images were analyzed using WinRHIZOTron software (Régent Instruments Inc., Quebec, QC, Canada) and presented as *L*a (mm cm^−2^; total root length per image area (3.24 cm^2^)).

Different soil horizons (0–20, 20–40, and 40–60 cm soil depth) were also collected during the harvesting stage in June 2015. Root samples from both treatments and across cultivars were carefully washed under a set of large to fine screens, roots were separated, and then root components were measured using a WinRHIZO image analysis system (V5.0, Regent Instruments, Quebec, QC, Canada).

### 4.5. Shoot Morphology, Leaf Physiology, and Yield

Parameters used as a measure of plant morphology (width and height) and their main physiological processes (LAI, SPAD, *Pn, g*s, and *E*) were determined during vegetative and harvesting stages in both growing seasons. Gas exchange (*Pn, g*s, and *E*) was measured using a portable photosynthesis system (LI-6400XT; LI-COR, Lincoln, NE, USA) following the procedures of Othman et al. [41]. Leaf-level gas exchange measurements were carried out between 11:00 a.m. and 1:00 p.m. on sun-exposed and fully matured leaves (2 leaves per replicate, two measurement per growing season) [42,43]. Light intensity was set to track ambient photosynthetically active radiation, area of chamber head to 6 cm^2^, flow rate to 500 μmol s^−1^, temperature in the cuvette to ambient air and reference CO_2_ to 390 μmol. SPAD was measured using a chlorophyll meter (SPAD-502 Plus, Minolta, Japan), and LAI was measured using a ceptometer (LP-80, Decagon Devices, Pullman, WA, USA). Artichoke harvests were conducted between April and May 2015 and 2016 and marketable yield (t ha^−1^) was determined. A head was considered marketable when its diameter was larger than 7 cm, without tipburn and/or open bracts [20]. In both growing seasons (2015 and 2016), a representative sample of eight heads per treatment was taken and used to measure phytochemical (chlorogenic acid and cynarin) concentration using a high-performance liquid chromatography system (HPLC, Waters Alliance 2695 Separation Module, Milford, MA 01757, USA).

### 4.6. Statistical Analysis

The study was designed using a randomized complete block design with four replications and two factors (two planting methods, three cultivars). The analysis of variance (ANOVA) and the least significant difference test (*p* < 0.05) in SAS (Version 9.4 for Windows; SAS Institute, Cary, NC, USA) were used to identify differences between planting methods (direct seeding vs. transplanting), cultivars, and their interactions.

## 5. Conclusions

Overall, the two-year field assessment of stand establishment methods, transplanting and direct seeding, revealed significant quantitative responses of root trait components, which were translated into yield differences between the two systems in the three cultivars evaluated. Transplants consistently exhibited increased *L*a, root length, and surface area, as well as shoot size, chlorophyll content index, and marketable yield. However, no significant differences were found in root diameter and volume, leaf-level gas exchange (*Pn*, *g*s, and *E*), and head cynarin concentration across the study period. Given that higher yield is the main concern for artichoke growers, this study supports that transplanting is the best growing method for globe artichoke cultivars. Since globe artichoke has one of the highest total antioxidant capacities among all vegetables, our future research will focus on how to couple the increase in artichoke yield with optimal concentrations of antioxidant compounds and enzymes in the heads. That research will provide a better understanding on the balance between yield promotion and phytonutrient quality, including the level of protection from oxidative stress in transplanted globe artichoke plants.

## Figures and Tables

**Table 1:** Mean root length intensity (*L*a, mm cm^−2^) of artichoke cultivars (Green Globe Improved (GGI), Romolo (ROM), and Imperial Star (IS)) as influenced by planting method (direct seeding—Seed, and transplanting—transplant) and soil depth during 2015 and 2016 seasons. Data were collected using the minirhizotron root system.

Main Effect	Root Length Intensity (*L*a, mm cm^−2^)										
	2015					2016					Overall Mean
	March	April	May	July	Mean	March	May	June	July	Mean	
**Planting method (P)**											
Seed	0.72 a	1.46 b	1.03 a	0.81 a	1.01 b	0.29 a	1.27 a	0.48 a	1.97 b	1.01 b	1.01 b
Transplant	0.87 a	2.54 a	1.22 a	1.12 a	1.43 a	0.49 a	1.32 a	0.55 a	7.22 a	2.40 a	1.92 a
Cultivar (C)											
GGI	0.53 b	1.01 b	1.40 a	1.26 a	1.07 b	0.21 b	1.91 a	0.08 c	8.06 a	2.56 a	1.82 a
ROM	0.71 a	2.63 a	1.39 a	1.04 ab	1.51 a	0.92 a	1.01 b	0.42 b	3.43 b	1.44 b	1.48 b
IS	0.56 a	2.27 a	0.60 b	0.59 b	1.08 b	0.06 c	0.95 b	1.05 a	2.31 c	1.09 b	1.01 c
Depth (D, cm)											
20	1.06 a	2.52 a	1.77 a	1.86 a	1.80 a	1.06 a	1.43 ab	0.66 a	3.91 cd	1.77 b	1.78 a
40	1.05 a	1.95 ab	0.78 b	0.93 b	1.18 b	0.39 b	1.92 a	0.69 a	6.34 a	2.33 a	1.76 a
60	1.01 a	2.17 a	1.08 b	0.90 b	1.29 b	0.33 bc	1.50 ab	0.51 a	5.66 ab	2.00 ab	1.65 a
80	0.68 a	1.95 ab	1.14 b	0.69 b	1.12 b	0.14 bc	1.17 b	0.43 a	4.51 bc	1.56 b	1.34 b
100	0.19 b	1.41 b	0.82 b	0.43 b	0.71 c	0.04 c	0.43 c	0.30 a	2.62 d	0.85 c	0.78 c
ANOVA											
P	NS	***	NS	NS	***	†	NS	NS	***	***	***
C	**	***	**	*	**	***	**	***	***	***	***
P×C	**	***	NS	**	***	***	*	**	***	**	NS
D	***	**	**	**	***	***	*	NS	***	**	***
P×D	NS	NS	NS	NS	NS	***	NS	NS	NS	NS	NS
C×D	**	***	***	NS	***	***	NS	NS	*	†	**
P×C×D	NS	NS	NS	*	*	***	NS	NS	NS	NS	**

†, *, **, *** show significant differences at *p* < 0.1, 0.05, 0.01, and 0.001, respectively. NS, not significant at *p* < 0.1. Means in columns followed by different letters are significantly different at *p* < 0.05.

**Table 2:** Root trait components of artichoke cultivars (Green Globe Improved (GGI), Romolo (ROM), and Imperial Star (IS)) as influenced by planting method (direct seeding—Seed, and transplanting—Transplant) and soil depth during the harvesting period, July 2015.

Main Effect	Length (cm)	Surface Area (cm^2^)	Average Diameter (mm)	Volume (cm^3^)
**Planting method (P)**				
Seed	136 b	10.6 b	0.32 a	0.10 a
Transplant	231 a	15.2 a	0.29 a	0.09 a
Cultivar (C)				
GGI	197 a	15.5 a	0.35 a	0.14 a
ROM	119 b	10.6 b	0.34 a	0.09 b
IS	235 a	12.6 b	0.22 b	0.06 b
Depth (D)				
20	330 a	20.0 a	0.22 b	0.10 b
40	107 b	13.0 b	0.36 a	0.15 a
60	114 b	5.6 c	0.33 a	0.04 c
ANOVA				
P	***	**	NS	NS
C	***	***	**	*
P×C	**	**	NS	*
D	***	***	***	***
P×D	***	***	NS	NS
C×D	***	***	NS	*
P×C×D	**	***	NS	**

*, **, *** show significant differences at *p* < 0.05, 0.01, and 0.001, respectively. NS, not significant at *p* < 0.05. Means in columns followed by different letters are significantly different at *p* < 0.05.

**Table 3:** Plant width and height, leaf area index (LAI), and chlorophyll content index (SPAD) of artichoke cultivars (Green Globe Improved (GGI), Romolo (ROM), and Imperial Star (IS)) as influenced by planting method (direct seeding—Seed, and transplanting—Transplant) during 2015 and 2016 seasons.

Year	Main Effect	Width (cm)		Height (cm)		Leaf Area Index		SPAD	
		Vegetative	Harvesting	Vegetative	Harvesting	Vegetative	Harvesting	Vegetative	Harvesting
**2015**	**Planting method (P)**								
	Seed	80.3 b	162 b	12.0 a	71.9 b	1.60 b	3.33 b	38.4 b	57.0 a
	Transplant	105 a	191 a	15.3 a	93.1 a	2.10 a	3.80 a	44.0 a	58.7 a
	Cultivar (C)								
	GGI	88.5 b	180 a	15.2 a	86.4 a	1.77 a	3.19 b	42.3 a	61.0 a
	ROM	97.2 a	178 a	14.6 a	82.5 a	1.94 a	3.73 a	39.8 a	56.2 a
	IS	92.1 ab	172 a	11.0 a	78.6 a	1.84 a	3.77 a	41.5 a	56.5 a
	ANOVA								
	P	*	**	NS	**	*	*	**	NS
	C	*	NS	NS	NS	NS	*	NS	NS
	P×C	NS	NS	NS	NS	NS	NS	NS	NS
**2016**	**Planting method**								
	Seed	67.2 b	160 a	18.3 b	73.5 a	1.4 a	5.25 b	56.7 a	60.0 a
	Transplant	89.1 a	167 a	21.8 a	77.8 a	1.5 a	5.86 a	57.2 a	61.4 a
	Cultivar								
	GGI	73.3 a	167 a	23.8 a	85.9 a	1.3 a	5.10 b	59.4 a	64.5 a
	ROM	90.7 a	174 a	20.1 ab	75.3 ab	1.6 a	6.62 a	56.4 ab	61.1 ab
	IS	70.5 a	150 b	16.3 b	65.7 b	1.4 a	4.95 b	55.0 b	56.6 b
	ANOVA								
	P	*	NS	*	NS	NS	**	NS	NS
	C	NS	***	**	*	NS	**	*	*
	P×C	NS	NS	NS	NS	NS	NS	NS	NS

*, **, *** show significant differences at *p* < 0.05, 0.01, and 0.001, respectively. NS, not significant at *p* < 0.05. Means in columns followed by different letters are significantly different at *p* < 0.05.

**Table 4:** Marketable yield, chlorogenic acid, and cynarin of artichoke cultivars (Green Globe Improved (GGI), Romolo (ROM), and Imperial Star (IS)) as influenced by planting method (direct seeding—Seed, and transplanting—Transplant) during 2015 and 2016 seasons.

Year	Main Effect	Marketable Yield (ton ha^−1^)	Chlorogenic Acid (µg g^−1^)	Cynarin (µg g^−1^)
**2015**	**Planting method (P)**			
	Seed	19.9 b	125 a	5.63 a
	Transplant	21.1 a	138 a	6.26 a
	Cultivar (C)			
	GGI	24.3 a	95 b	5.65 a
	ROM	21.9 b	140 ab	6.65 a
	IS	15.2 c	159 a	5.53 a
	ANOVA			
	P	*	NS	NS
	C	*	*	NS
	P×C	NS	NS	NS
**2016**	**Planting method**			
	Seed	13.7 b	312 a	6.70 a
	Transplant	18.3 a	144 b	6.23 a
	Cultivar			
	GGI	17.5 a	241 ab	6.76 a
	ROM	15.1 b	284 a	6.21 a
	IS	15.5 ab	175 b	6.52 a
	ANOVA			
	P	***	***	NS
	C	*	*	NS
	P×C	NS	NS	NS

*, *** show significant differences at *p* < 0.05 and 0.001, respectively. NS, not significant at *p* < 0.05. Means in columns followed by different letters are significantly different at *p* < 0.05.
